# A deep learning algorithm to identify carotid plaques and assess their stability

**DOI:** 10.3389/frai.2024.1321884

**Published:** 2024-06-17

**Authors:** Lan He, Zekun Yang, Yudong Wang, Weidao Chen, Le Diao, Yitong Wang, Wei Yuan, Xu Li, Ying Zhang, Yongming He, E. Shen

**Affiliations:** ^1^Department of Ultrasound Medicine, Shanghai Chest Hospital, Shanghai Jiao Tong University School of Medicine, Shanghai, China; ^2^Department of Ultrasound Medicine, Shanghai Eighth People’s Hospital, Shanghai, China; ^3^Infervision, Beijing, China; ^4^Department of Ultrasound Medicine, Xinhua Hospital, Dalian University, Dalian, China; ^5^Department of Ultrasound Medicine, Caohejing Street Community Health Service Centre, Shanghai, China; ^6^Department of Cardiology, The First Hospital of Soochow University, Suzhou, China

**Keywords:** deep learning, carotid plaque stability, ultrasound, convolutional neural network, BCNN-ResNet algorithms

## Abstract

**Background:**

Carotid plaques are major risk factors for stroke. Carotid ultrasound can help to assess the risk and incidence rate of stroke. However, large-scale carotid artery screening is time-consuming and laborious, the diagnostic results inevitably involve the subjectivity of the diagnostician to a certain extent. Deep learning demonstrates the ability to solve the aforementioned challenges. Thus, we attempted to develop an automated algorithm to provide a more consistent and objective diagnostic method and to identify the presence and stability of carotid plaques using deep learning.

**Methods:**

A total of 3,860 ultrasound images from 1,339 participants who underwent carotid plaque assessment between January 2021 and March 2023 at the Shanghai Eighth People’s Hospital were divided into a 4:1 ratio for training and internal testing. The external test included 1,564 ultrasound images from 674 participants who underwent carotid plaque assessment between January 2022 and May 2023 at Xinhua Hospital affiliated with Dalian University. Deep learning algorithms, based on the fusion of a bilinear convolutional neural network with a residual neural network (BCNN-ResNet), were used for modeling to detect carotid plaques and assess plaque stability. We chose AUC as the main evaluation index, along with accuracy, sensitivity, and specificity as auxiliary evaluation indices.

**Results:**

Modeling for detecting carotid plaques involved training and internal testing on 1,291 ultrasound images, with 617 images showing plaques and 674 without plaques. The external test comprised 470 ultrasound images, including 321 images with plaques and 149 without. Modeling for assessing plaque stability involved training and internal testing on 764 ultrasound images, consisting of 494 images with unstable plaques and 270 with stable plaques. The external test was composed of 279 ultrasound images, including 197 images with unstable plaques and 82 with stable plaques. For the task of identifying the presence of carotid plaques, our model achieved an AUC of 0.989 (95% CI: 0.840, 0.998) with a sensitivity of 93.2% and a specificity of 99.21% on the internal test. On the external test, the AUC was 0.951 (95% CI: 0.962, 0.939) with a sensitivity of 95.3% and a specificity of 82.24%. For the task of identifying the stability of carotid plaques, our model achieved an AUC of 0.896 (95% CI: 0.865, 0.922) on the internal test with a sensitivity of 81.63% and a specificity of 87.27%. On the external test, the AUC was 0.854 (95% CI: 0.889, 0.830) with a sensitivity of 68.52% and a specificity of 89.49%.

**Conclusion:**

Deep learning using BCNN-ResNet algorithms based on routine ultrasound images could be useful for detecting carotid plaques and assessing plaque instability.

## 1 Introduction

The incidence of stroke exceeds 100 per 100,000 and is increasing year by year. And because the death rate from stroke is close to 300 per 100,000, the high morbidity and mortality rates make the cost of stroke treatment among the highest (Hu et al., 2020). Up to one quarter of strokes can be attributed to the rupture and shedding of unstable carotid plaques (Murray et al., 2018; Sun et al., 2018; Parish et al., 2019; Kopczak et al., 2020). Plaque stability is commonly based on the following features: shape, structure, lipids, fibrous caps, and calcification (Baradaran and Gupta, 2021; Bos et al., 2021).

Traditional carotid plaque identification relies on obtaining images of patients’ necks through techniques such as ultrasound examination (Murray et al., 2018), computed tomography (CT) (Zhang et al., 2022), magnetic resonance imaging (MRI) (Hajhosseiny et al., 2019) and so on, which are then analyzed by experienced doctors for diagnosis. These imaging techniques are categorized into invasive and non-invasive methods. To reduce the burden on patients and to acquire the necessary images more rapidly, ultrasound examination, known for its non-invasive nature and capability for real-time imaging, is widely used in clinical settings (Tjoa and Guan, 2021). Due to factors like the uneven distribution of medical resources across different regions and the inherent limitations of ultrasound imaging technology, ultrasound images are prone to a significant amount of noise. The diagnostic process conducted by doctors can be influenced by variables such as the expertise of the physician, their knowledge background, fatigue, and other factors. When combined with the interference from these noises, subjective bias can occur in the diagnostic outcomes.

Before the application of deep learning to carotid plaque identification, researchers often used methods such as edge detection algorithms (Kerwin et al., 2007), region-growing algorithms (Francois et al., 2011), and texture-based analysis for plaque detection (Molinari et al., 2007). However, edge detection algorithms and region-growing algorithms are limited by the image quality and are particularly sensitive to changes in brightness and color. Texture-based analysis methods can be significantly affected by the high-density noise present in ultrasound images.

Therefore, we believe that these challenges can be solved through automated analysis through deep learning (Wang et al., 2021), which has already shown great promise in medical image analysis, spanning from screening, diagnosing to prognosis prediction in varying disease such as lung cancer, skin cancer, and breast cancer (Chaudhary et al., 2018; Roy-Cardinal et al., 2019; Ying et al., 2022; Wang et al., 2023). Machine learning could detect carotid plaques (Chen et al., 2021), but requires manual segmentation. Deep learning, in contrast, automates feature extraction for potentially greater reliability and accuracy (LeCun et al., 2015).

Among the various deep learning algorithms reported so far, Convolutional Neural Networks (CNNs) (Wang and Qi, 2023) such as VGG-16, ResNet (Johri et al., 2020) and their variants have shown particularly good performance in image feature classification. Therefore, we attempted to directly apply these networks to the task of plaque recognition and classification in ultrasound images. However, these traditional network models seem to struggle with processing conventional ultrasound images, which are often filled with noise, especially where plaques usually occupy a relatively small area. Conventionally, increasing the number of network parameters theoretically benefits the model’s fitting effect. We started testing with ResNet-18 and found that ResNet-50, which has more parameters, improved the model’s performance. Consequently, we explored using bilinear CNNs on top of Resnet to increase the network’s parameters without increasing its depth, providing a more detailed representation of ultrasound image features. This approach makes the algorithm more robust to heterogeneity and noise in images. The ability to use image features can enhance supervised information, aiding in improving the proficiency of image classification.

Therefore, the objective of this study is to develop and validate a novel BCNN-ResNet that facilitates automated detection of carotid plaques and assessment of their instability from routine ultrasound images, thereby aiding in the efficient screening and prevention of stroke associated with carotid artery disease.

## 2 Dataset and methods

### 2.1 Data and quality control

#### 2.1.1 The BCNN-ResNet dataset

Doppler ultrasound images of the bilateral carotids, which underwent health checkup and carotid plaque screening. A total of 3,860 ultrasound images from 1,339 participants who underwent carotid plaque assessment between January 2021 and March 2023 at the Shanghai Eighth People’s Hospital were divided into a 4:1 ratio for training and internal testing. The external test included 1,564 ultrasound images from 674 participants who underwent carotid plaque assessment between January 2022 and May 2023 at Xinhua Hospital affiliated with Dalian University. Participants were excluded from the study if longitudinal ultrasound images were unavailable, annotated measurement size markers, insufficient quality. At last, Modeling for detecting carotid plaques involved training and internal testing on 1,291 ultrasound images, with 617 images showing plaques and 674 without plaques. The external test comprised 470 ultrasound images, including 321 images with plaques and 149 without. Modeling for assessing plaque stability involved training and internal testing on 764 ultrasound images, consisting of 494 images with unstable plaques and 270 with stable plaques. The external test was composed of 279 ultrasound images, including 197 images with unstable plaques and 82 with stable plaques. Details see Supplementary Figure S1.

#### 2.1.2 Carotid plaques ultrasound imaging quality control

**Figure 1:**
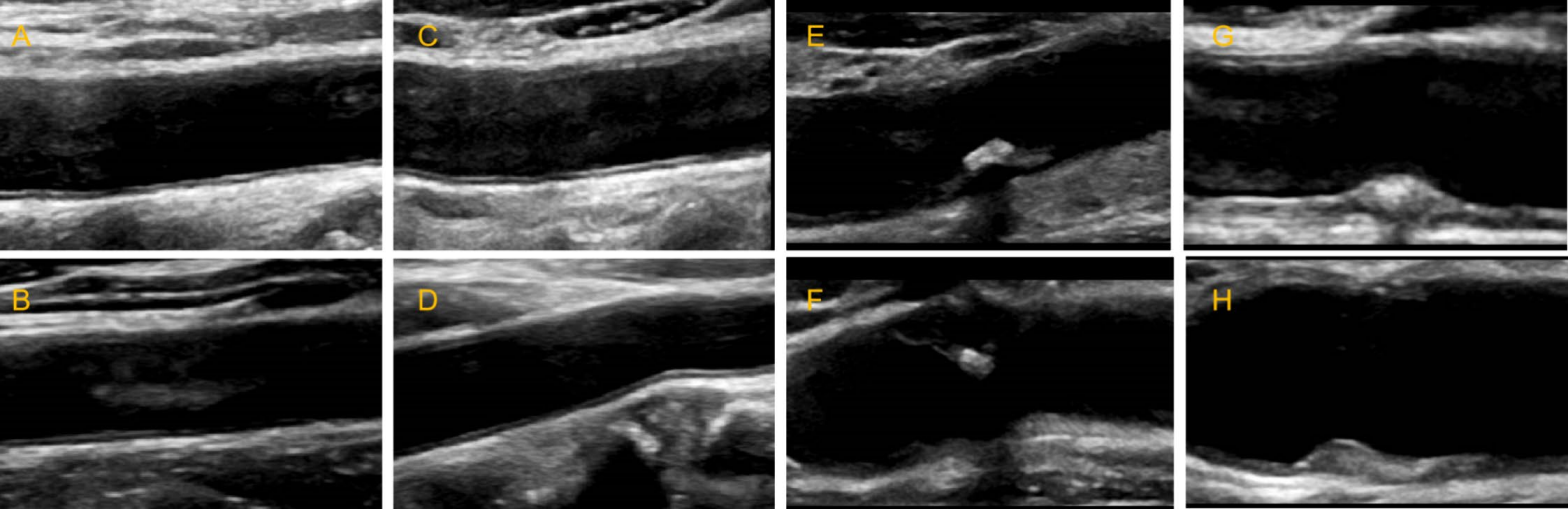
Schematic diagram of carotid ultrasound image retention standards. **(A–D)** The normal carotid. **(E-H)** The carotid plaques.

Carotid plaques and stability were assessed using longitudinal scanning images based on the 2020 American Society of Echocardiography guidelines (Zhou et al., 2021). The presence of plaques were defined as deposits with intima-media thickness (IMT) ≥1.5 mm or that protruded into the lumen or whose thickness exceeded 50% of the peripheral IMT. Plaques were considered stable if their morphology was regular in shape, their echogenicity was uniform, and their surface was smooth and continuous with an intact fibrous cap. Plaques were considered unstable when they exhibited inordinate morphology, uneven echogenicity, a thin fibrous cap that was incomplete or ruptured, a hypoechoic plaque or ulcerated plaque that revealed damaged areas on the plaque surface, or lipid cores that covered more than 40% of the plaque area. Color Doppler ultrasonography was conducted with a 7.5–12.0 MHz probe on patients in a supine position. Five imaging systems were used: EPIC7c (Philips, Amsterdam, Netherlands), Affiniti70 (Philips, Amsterdam, Netherlands), *Aloka* ARIETTA 60 (HITACHI, Tokyo, Japan), GEs8 (GE, Fairfield, Connecticut, United States), Aplio400 (TOSHIBA, Tokyo, Japan). The probe was longitudinally scanned perpendicularly to the neck in order to maintain a scan depth of 3–4 cm. The carotid was placed in the middle of the image in the case of patients diagnosed with a normal artery; otherwise, the plaque was placed in the middle of the image (show as Figure 1). During scanning, gain was 50–70 dB; power, 40–50 Hz; and the angle between the flow beam and the speed of sound, ≤60°. One or two images in both DICOM and JPG formats, without measurement size markers, were analyzed for each participants. Resolution was standardized across all images to be 256 × 256 pixels.

Using Wei Ning Ultrasound Information Management System (miis60pro, Hefei, China), four experienced physicians analyzed images for the presence or absence of plaques and classified them as stable or unstable. The images and assessments were reviewed by two chief physicians who resolved disagreements through discussion.

### 2.2 Deep learning models

#### 2.2.1 Image preprocessing

We first manually cropped all images to a standard size in order to remove irrelevant background areas and retain only the ultrasound image region. We then normalized the data using the mean and standard deviation of all images in order to ensure that data followed a standard distribution.

To enhance the model’s ability to generalize and improve its robustness, we employed data augmentation techniques such as random horizontal flipping, random vertical flipping, and random rotation. In order to mitigate the overfitting issue, which could arise due to the presence of a small number of erroneous samples, we incorporated label smoothing into our strategy. This approach prevented the model from relying excessively on training samples, ensuring a more balanced learning process.

#### 2.2.2 Development of the BCNN-ResNet algorithm

The BCNN-ResNet algorithm is designed to construct a model that includes autonomously cropping each image to leave only the carotid artery and potential plaques, as well as for the identification and classification of plaques. Before building this algorithm, we attempted to start with various existing CNN models and Transformer models, training them with our dataset. Due to the small size of the dataset, models with Transformer architecture performed poorly on this task. Among the common CNN models, the ResNet network was superior in feature extraction for ultrasound images, which are full of high-frequency noise, compared to other CNN models like VGG. Analyzing the feature heatmaps of the ResNet model, we found it had a significant advantage in recognizing the vascular system within ultrasound images (Lei et al., 2020; Cheng et al., 2022; Johri et al., 2022; Shokouhmand et al., 2023), where plaques are located.

Starting with ResNet-18 for this task, we found that ResNet-50, which has more parameters, improved the model’s performance, but the performance of ResNet-34 actually worsened. Therefore, we sought a method to increase the number of model parameters without increasing the model’s depth. Thus, we proposed the BCNN-ResNet network, using ResNet-50 as the backbone network, linearly combining features extracted by two ResNet-50 networks. Feature fusion is achieved by bilinearly combining two images of the same resolution as the original image, creating a mixed feature matrix that aligns pixels at corresponding positions in both images. The fused features are then input into a fully connected layer, which outputs the classification results. The first ResNet-50 network takes edge-extracted ultrasound images as input, retaining only the structural information of the image. The second ResNet-50 network uses the original ultrasound images as input to extract pixel information. The fusion of structural and pixel information enables finer image classification.

#### 2.2.3 Modeling for detecting carotid plaques

**Figure 2:**
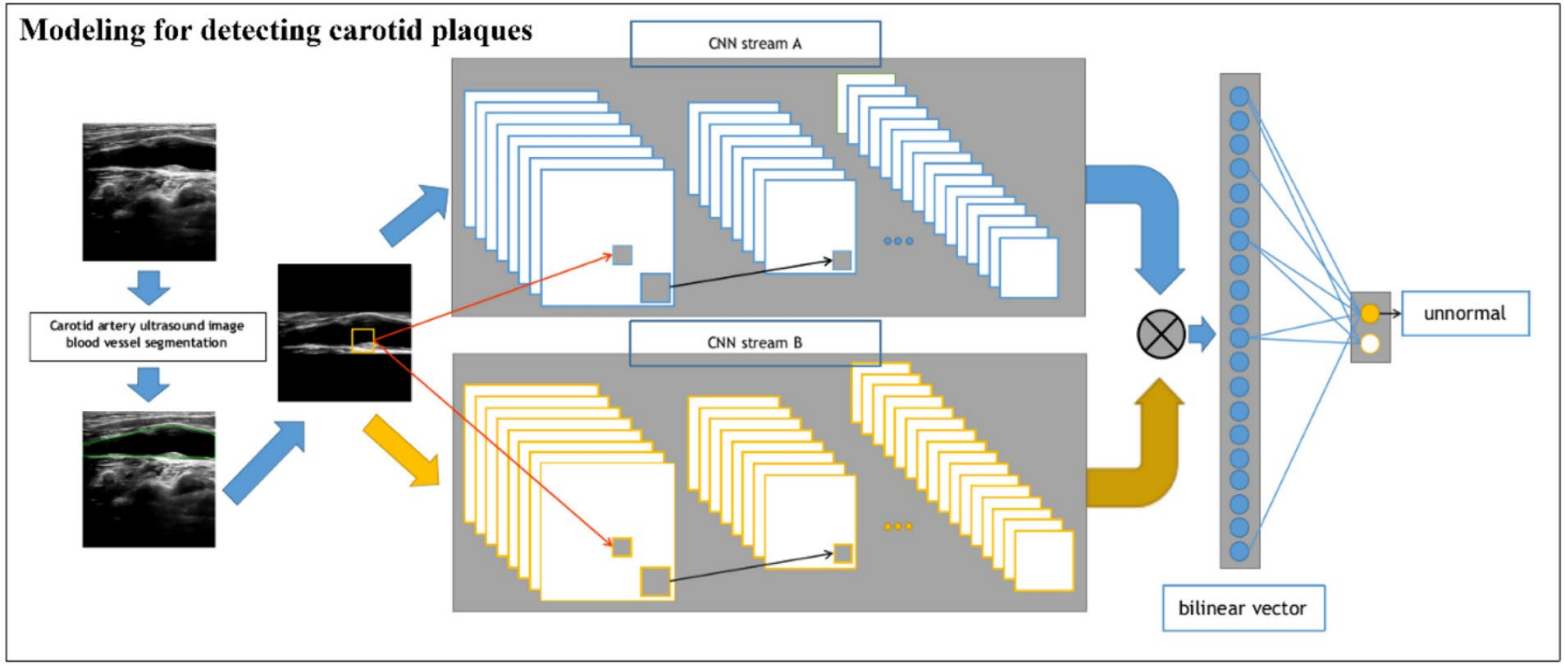
Schematic of the BCNN-ResNet algorithm. Flowchart of carotid plaque detection using a single-input algorithm.

For the classification network of plaque presence and absence (Figure 2), we built a BCNN network using two Res-Net50 networks but only a single input. Let the two networks perform feature extraction on the image separately, and then bilinear feature combination of the two features at the same location, stretch the mixed feature matrix into a vector, and perform moment normalization and L2 normalization on the vector to obtain the fused features, and finally use the fully connected layer for classification.

#### 2.2.4 Modeling for assessing plaque stability

**Figure 3:**
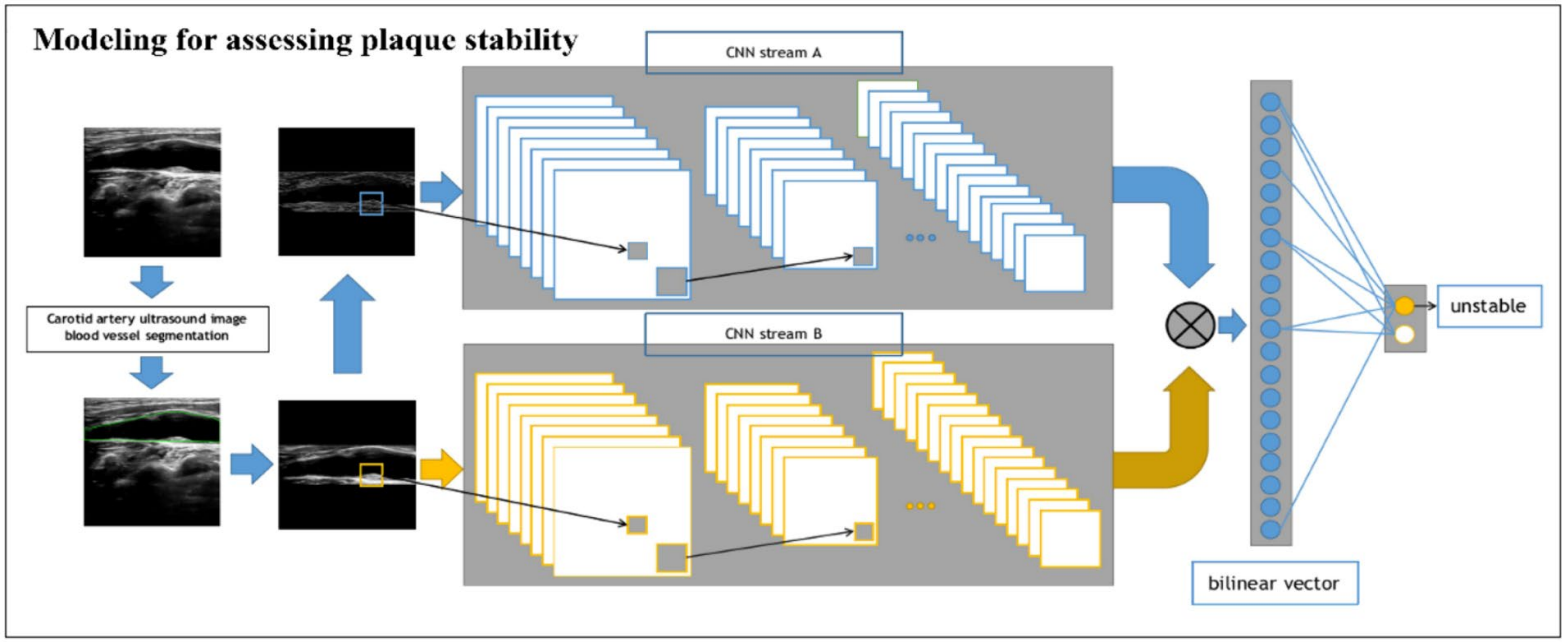
Schematic of the BCNN-ResNet algorithm. Flowchart of plaque stability assessment using a dual-input algorithm.

For the stability classification task of plaques (Figure 3), we build a dual-input BCNN network on top of the single-input BCNN network to extract more obvious difference features to improve the model effect since the pixel difference between different plaques is smaller than that of the previous task. The dual-input network still uses two Res-Net50 networks as the backbone network, but has two inputs, which are ultrasonic image after edge extraction retaining only the structural information of the image; the second one uses the original ultrasound image as the input to extract the pixel information to complete the finer-grained image classification task. A detailed processing flowchart of the Model 2 network in Supplementary Figure S2.

#### 2.2.5 Parameter setting

We trained the BCNN-ResNet algorithm using modeling for detecting carotid plaques training dataset and modeling for assessing plaque stability training dataset, the AdamW optimization algorithm and a batch size of 64, while dynamic learning rate adjustments were made through cosine annealing. Before we decided to use ResNet-34 as the backbone network, we tested whether to use pre-trained weights. The ResNet-34 network using completely random initialization parameters lags behind the AUC indicator by about 4% compared to the ResNet34 network pre-trained using imagenet. Therefore, the backbone network of two ResNet-34 models was initialized using pre-trained ImageNet parameters. We further refined the cross-entropy loss function to incorporate label smoothing, thereby enhancing model robustness. The algorithm was executed within the PyTorch machine learning framework. Training was performed on four Nvidia RTX 4000 GPUs.

### 2.3 Algorithm evaluation

#### 2.3.1 Evaluation modeling for detecting carotid plaques and modeling for assessing plaque stability algorithms

Modeling for detecting carotid plaques and Modeling for assessing plaque stability algorithms were validated using an internal testing dataset from patients belonging to the same cohort as those used for training, as well as an external testing dataset from patients who were non-overlap with the training and internal testing dataset. In both cases, algorithm performance was assessed for the two tasks of plaque detection and plaque stability assessment in terms of the area under the AUC (the area under the ROC curve), accuracy, sensitivity, and specificity. Thresholds for defining whether a plaque was present or absent or whether it was stable or unstable were optimized using the Youden index. Where appropriate, results were reported with 95% confidence intervals (CIs).

#### 2.3.2 Demonstration of modeling for assessing plaque stability algorithms using gradient-weighted class activation mapping

In order to describe the prediction from our Modeling for assessing plaque stability, we used Grad-CAM network visualization methods (Libby et al., 2019) to generate model’s heat-maps. Higher intensity areas in a heat-map correspond to regions in the input image that contribute more to the model’s prediction. These areas are where the model focused its attention during the classification process. Conversely, lower intensity areas are less influential. Our heat-maps are the same as most Grad-CAM heat-maps, using a color scale to represent the intensity of importance. Warmer colors like red and yellow indicate higher importance, while cooler colors like blue and green represent lower importance.

#### 2.3.3 Statistical analyses

Continuous data showing a normal distribution were reported as mean ± standard deviation, and inter-group differences were assessed for significance using a Student’s *t* test. Continuous data showing a skewed distribution were reported as median (interquartile range), and inter-group differences were assessed using a non-parametric test such as the Mann–Whitney U test.

Categorical data were reported as *n* (%), and inter-group differences were assessed using a chi-squared test if *n* > 40 or Fisher’s exact test otherwise.

#### 2.3.4 Ethics statement

The study was approved by the Ethics Review Board at Shanghai Eighth People’s Hospital (approval 2022-015-09-02) and the Xinhua Hospital affiliated with Dalian University (approval 2022-100-01). The requirement for informed consent was waived by the Ethics Review Boards.

## 3 Results

### 3.1 The study cohort

Modeling for detecting carotid plaques consisted 1761 ultrasound images from 1,165 participants, 510 with plaques, 655 without carotid plagues. Modeling for assessing plaque stability consisted 1,043 ultrasound images from 510 participants, 156 with stable plagues, 354 with unstable plagues.

Among the 1,165 participants included in the Modeling for detecting carotid plaques analysis, those with carotid plaques showed significantly higher Male, Age, Lipoprotein a, SBP, DBP, Uric acid, Apolipoprotein A1, Apolipoprotein B and significantly lower Apolipoprotein E, HDL-C, LDL-C, Total cholesterol than those without plaques (Table 1). Among the 510 participants included in the Modeling for assessing plaque stability final analysis, those with stable plaques showed significantly higher SBP, Apolipoprotein A1, Apolipoprotein E, HDL-C, Total cholesterol than those with unstable ones (Table 2).

**Table 1:** Clinicodemographic comparison between patients with or without plaques in the modeling for detecting carotid plaques.

Characteristic	No plaques (*n* = 655)	Plaques (*n* = 510)	*p*
Sex			<0.0001
Male	121 (18.47)	265 (51.96)	
Female	534 (81.53)	245 (48.04)	
Age, yr	57.15 ± 10.98	68.09 ± 12.46	<0.0001
Marital status			0.0299
Married	643 (98.17)	506 (99.22)	
Unmarried	12 (1.83)	4 (0.78)	
Triglycerides, mmol/L	1.42 (1.12, 1.75)	1.37 (0.960, 1.647)	0.0001
Lipoprotein a, mg/dL	148.50 (57.00, 188.70)	178.750 (65.10, 203.48)	<0.0001
SBP, mmHg	148.14 ± 31.58	157.02 ± 25.70	<0.0001
DBP, mmHg	80.09 ± 6.61	83.84 ± 8.31	<0.0001
Heart rate, bpm	77.41 ± 11.09	78.18 ± 10.15	0.2204
Uric acid, μmol/L	330.78 ± 73.78	353.57 ± 89.84	<0.0001
Apolipoprotein A1, g/L	1.21 ± 0.18	1.23 ± 0.18	<0.0001
Apolipoprotein B, g/L	0.79 ± 0.18	0.83 ± 0.20	0.001
Apolipoprotein E, g/L	42.34 ± 9.86	39.63 ± 9.14	<0.0001
HDL-C, mmol/L	1.21 ± 0.29	1.16 ± 0.26	0.0009
LDL-C, mmol/L	2.99 ± 0.79	2.84 ± 0.81	0.0016
Total cholesterol, mmol/L	4.86 ± 0.67	4.53 ± 0.93	<0.0001

Values are *n* (%), mean ± SD or median (interquartile range), unless otherwise noted. SBP, systolic blood pressure; DBP, diastolic blood pressure; HDL-C, high-density lipoprotein cholesterol; LDL-C, low-density lipoprotein cholesterol.

**Table 2:** Clinical characteristics of patients for stable and unstable plaques in the modeling for assessing plaque stability.

Characteristics	Stable, *N* = 156	Unstable, *N* = 354	*p* value
Sex			0.0028
Male	65 (41.67)	200 (56.50)	
Female	91 (58.33)	154 (43.50)	
Age, yr	67.26 ± 11.32	68.45 ± 12.93	0.3212
Marital history			0.1586
Married	155 (99.36)	350 (98.87)	
Unmarried	0 (0.00)	4 (1.13)	
Triglycerides, mmol/L	1.40 (1.07, 155)	1.37 (0.96, 1.66)	0.6224
Lipoprotein a, mg/dL	202.18 (74.00, 202.18)	172.00 (62.20, 207.00)	0.0544
SBP, mmHg	169.47 ± 32.55	151.61 ± 19.73	<0.0001
DBP, mmHg	83.60 ± 8.36	83.94 ± 8.29	0.6751
Heart rate, bpm	78.17 ± 7.66	78.20 ± 11.08	0.978
Uric acid, μmol/L	358.60 ± 80.34	351.36 ± 93.76	0.4021
Apolipoprotein A1, g/L	1.15 ± 0.17	1.12 ± 0.19	0.0429
Apolipoprotein B, g/L	0.86 ± 0.22	0.81 ± 0.19	0.0171
Apolipoprotein E, g/L	41.44 ± 9.17	38.84 ± 9.03	0.003
HDL-C, mmol/L	1.22 ± 0.21	1.13 ± 0.28	0.0002
LDL-C, mmol/L	2.90 ± 0.82	2.82 ± 0.80	0.3113
Total cholesterol, mmol/L	4.76 ± 0.91	4.43 ± 0.91	0.0002

Values are *n* (%), mean ± SD or median (interquartile range), unless otherwise noted. SBP, systolic blood pressure; DBP, diastolic blood pressure; HDL-C, high-density lipoprotein cholesterol; LDL-C, low-density lipoprotein cholesterol.

### 3.2 Model performance in detection of carotid plaques

**Figure 4:**
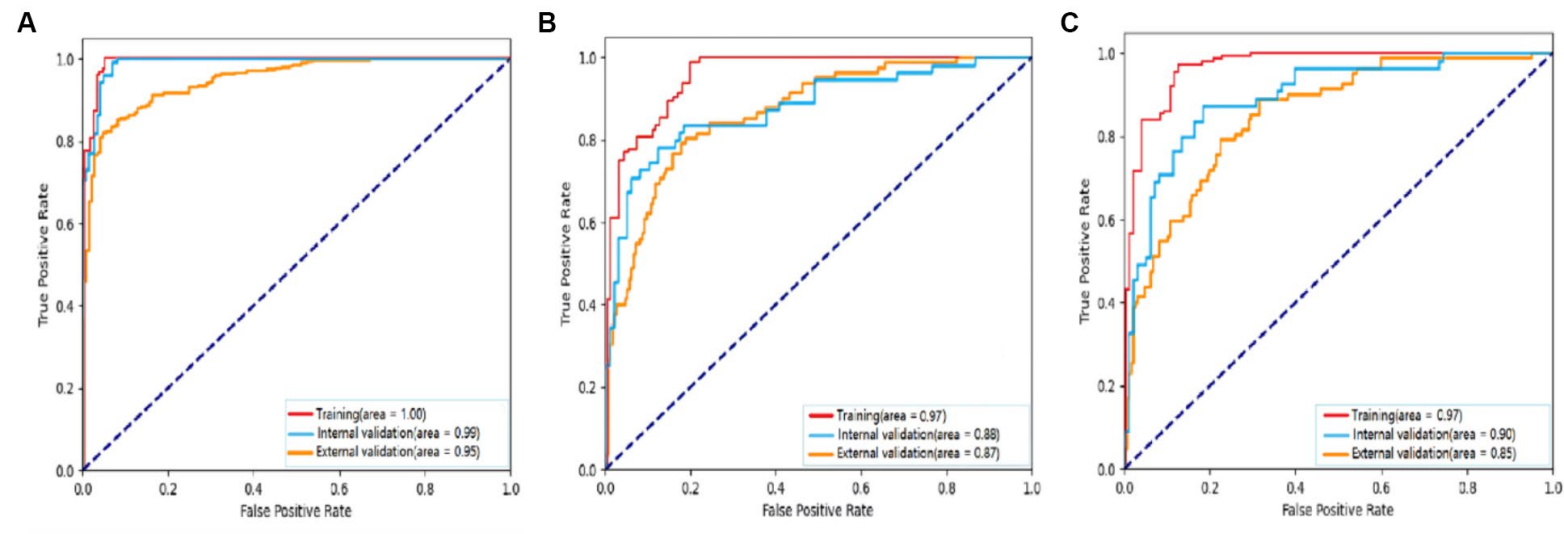
Receiver operating characteristic curves to assess the ability of the BCNN-ResNet algorithm to **(A)** detect carotid plaques or **(B,C)** assess plaque stability in **(B)** single-input or **(C)** dual-input mode. The algorithm was assessed against the training dataset (red curves), internal testing dataset (blue) or external testing dataset (gold). The area under each curve is indicated in the legends at the bottom right of each panel.

**Figure 5:**
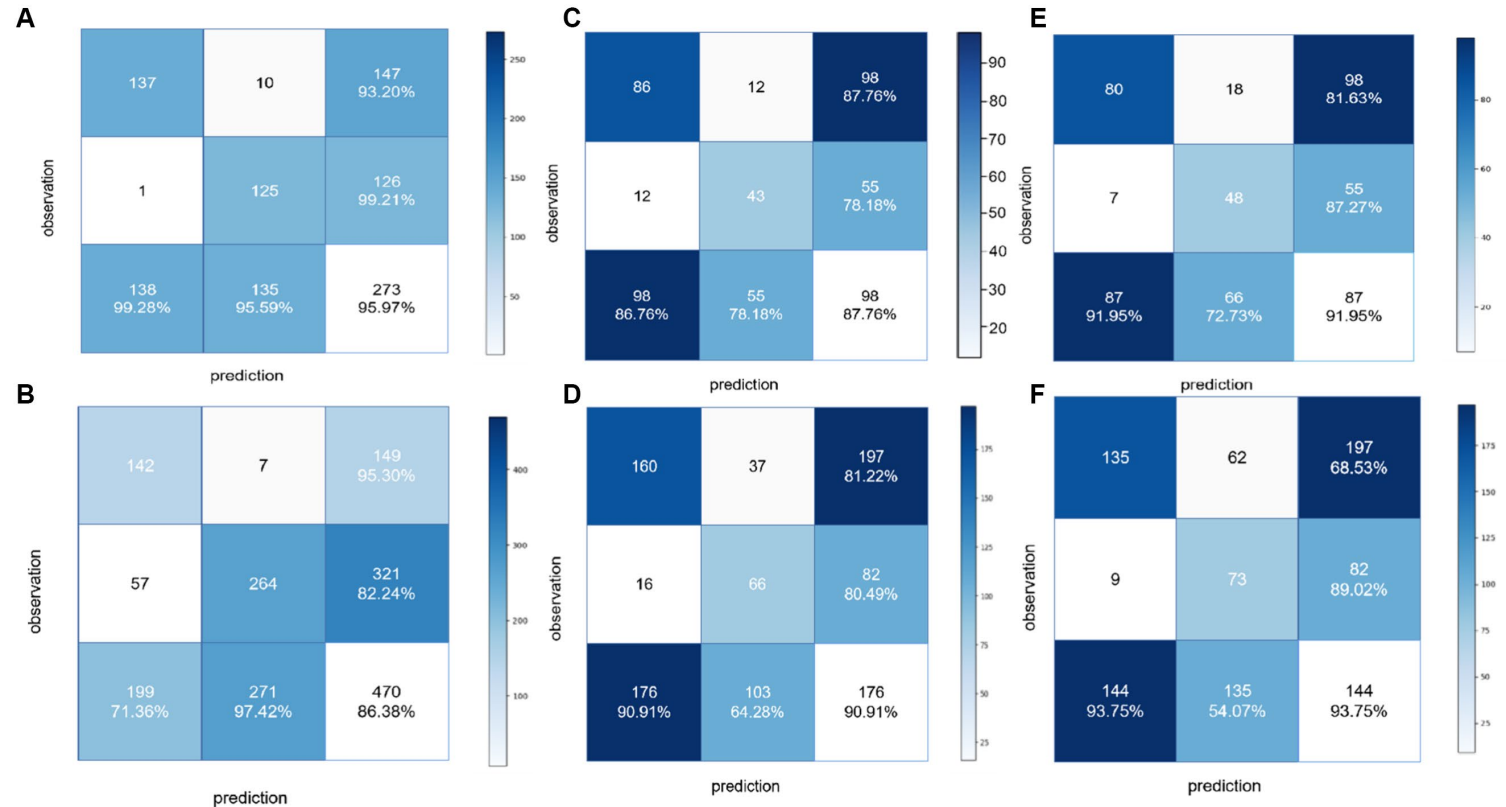
Confusion matrices to assess the ability of the BCNN-ResNet algorithm to **(A,B)** detect carotid plaques in the **(A)** training dataset or **(B)** external testing dataset; **(C,D)** assess plaque stability in single-input mode in the **(C)** training dataset or **(D)** external testing dataset; or **(E,F)** assess plaque stability in dual-input mode in the **(E)** training dataset or **(F)** external testing dataset.

For detecting carotid plaques in the internal testing dataset, the BCNN-ResNet algorithm had an AUC of 0.989 (95% CI 0.998–0.840), accuracy of 95.97%, sensitivity 93.20% and specificity 99.21%. In the external testing dataset, the AUC of 0.951 (95% CI 0.962–0.939), with 86.38% accuracy, 95.30% sensitivity and 82.24% specificity (Figures 4A, 5A,B and Table 3).

**Table 3:** Performance of BCNN-ResNet algorithms in internal and external testing.

Dataset	AUC (95% CI)	TP	FP	Accuracy (%)	Sensitivity (%)	Specificity (%)
Detection of carotid artery plaques						
Internal testing	0.989 (0.908, 0.840)	137	1	95.97	93.20	99.21
External testing	0.951 (0.962, 0.939)	142	57	86.38	95.30	82.24
Assessment of carotid plaque stability (single-input algorithm)						
Internal testing	0.878 (0.908, 0.840)	86	12	84.31	87.76	78.18
External testing	0.869 (0.893, 0.841)	160	16	81.00	81.22	80.49
Assessment of carotid plaque stability (dual-input algorithm)						
Internal testing	0.896 (0.922, 0.865)	80	7	83.66	81.63	87.27
External testing	0.854 (0.889, 0.830)	135	9	74.55	68.52	89.49

AUC, area under the receiver operating characteristic curve; CI, confidence interval; FP, false positive; TP, true positive.

### 3.3 Model performance in assessment of carotid plaques stability

For assessing plaques as stable or unstable, the performance parameters of AUC 0.896 (95% CI 0.922–0.865), accuracy of 83.66%, sensitivity of 81.63% and specificity of 87.27% in internal testing dataset, and 0.854 (95% CI 0.889–0.830), 74.55, 68.52 and 89.49% in the external testing dataset (Figures 4B,C, 5C–F and Table 3). The combination of BCNN and Res-Net50, especially in dual-input mode, assessed plaque stability markedly better than other Res-Net architectures (Supplementary Table S1).

**Figure 6:**
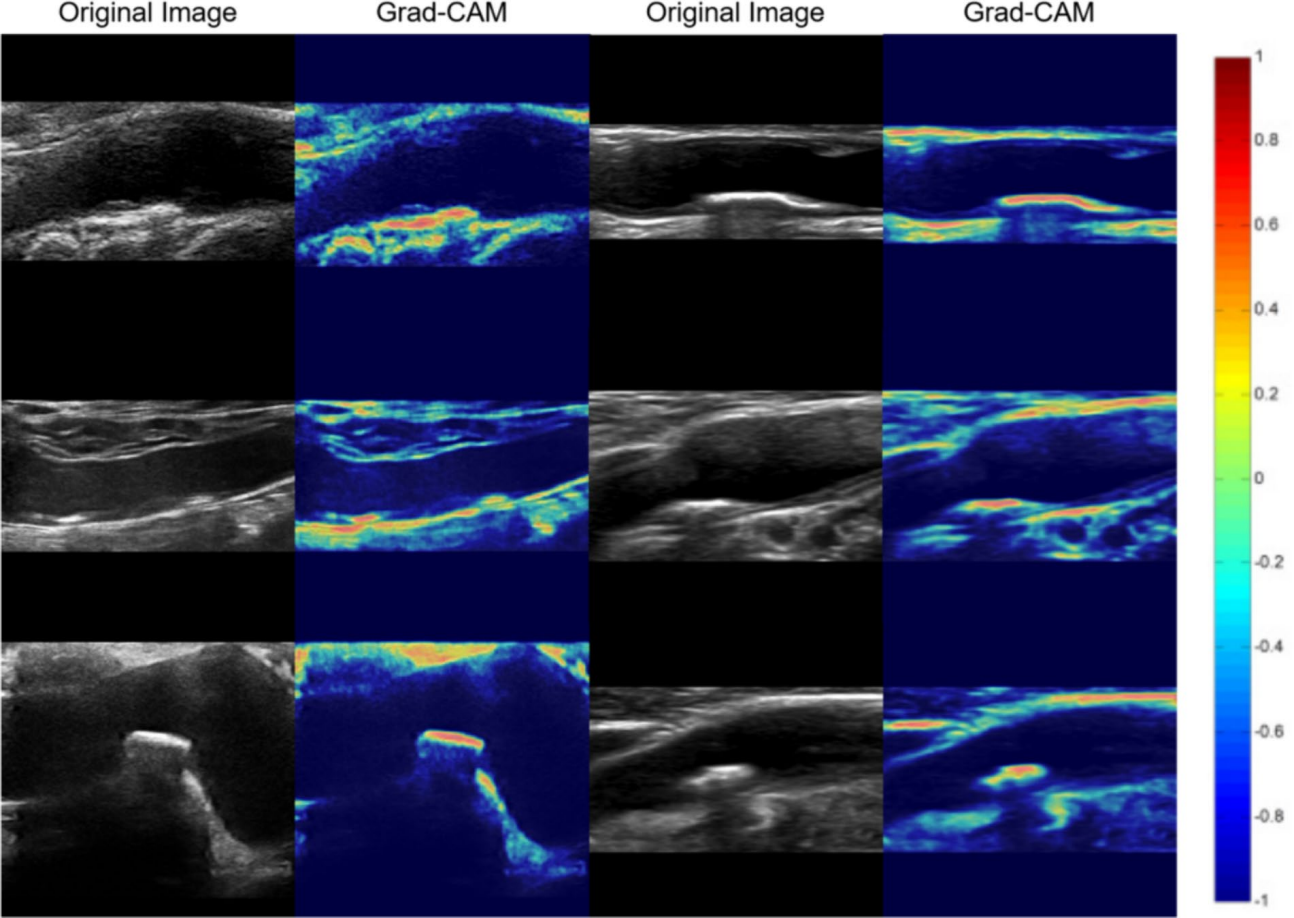
Gradient-weighted class activation mapping to identify areas of ultrasound images that the BCNN-ResNet algorithm weighed more during its calculations. Original ultrasound images that served as input are shown in grayscale, while the neighboring image is colored according to where the algorithm “focused.”

Original ultrasound images and gradient-weighted class activation maps from representative patients within Modeling for assessing plaque stability are shown in Figure 6.

## 4 Discussion

In this study, we built Modeling for detecting carotid plaques and Modeling for assessing plaque stability algorithms, we provide evidence that a novel combination of a BCNN and Res-Net architecture can accurately detect carotid plaques in ultrasound images, as well as assess their stability, which correlates with risk of stroke and other cardiovascular disorders (Selwaness et al., 2016; Saba et al., 2019, 2022; Van Der Toorn et al., 2022). Modeling for detecting carotid plaques and modeling for assessing plaque stability demonstrate excellent diagnostic performance. The proposal of this study provides a new and objective automatic quantitative approach for the detection of carotid plaque and the determination of plaque stability, which may provide an effective examination method for early screening of stroke in clinical practice.

For the ultrasound images of carotid, the ultrasound imaging effect has more noise compared with CT and other modalities, and the area of the plaque is smaller compared with the whole carotid image, the finer-grained image classification task, which is more suitable for the carotid ultrasound image classification task research. Our study justifies further work to optimize and develop the combined network into a tool for automated detection and analysis of carotid plaques, and it may help guide future efforts to integrate deep learning into complex image classification pathways in the clinic.

Our Modeling for detecting carotid plaques, the internal testing AUC was 0.989 (95% CI 0.998–0.840) and the external testing AUCwas 0.951 (95% CI 0.962–0.939), better than an AUC of 0.935 in previously reported deep learning method to identify plaque location (Li et al., 2021). A deep learning method has also been reported for assessing plaque area (Yu et al., 2021; Jain et al., 2022; Lin et al., 2022), which could help monitor plaque progression and regression. In our study, we identified the presence or absence of plaque in the carotid based on further identification of plaque stability, which is more clinically useful for clinical prediction of stroke than identifying the location and size of plaque, and unstable high-risk carotid plaque suggests a higher risk of stroke occurrence. Modeling for assessing plaque stability algorithms, the internal testing AUC (0.896), the external testing AUC (0.854), the internal testing AUC was better than previous existing models AUC (0.868) (We validated using the Modeling for Assessment Plaque stability dataset, see Supplementary Table S1). Our work, together with those previous studies, supports the idea that deep learning can accurately and reproducibly identify even small, subtle features in noisy ultrasound images. Indeed, deep learning appears to be superior to traditional machine learning algorithms for fine-grained interpretation of medical images (Biswas et al., 2021; Latha et al., 2022; van Dam-Nolen et al., 2022). In addition, machine learning requires more time and human involvement (van Veelen et al., 2022), for example, features need to be manually defined, which can make the algorithm’s classifications less reliable and robust to noise or patient heterogeneity. This observation provides a crucial understanding of the specific regions within the ultrasound imagery that significantly contribute to the predictions of the network. The aforementioned insight underscores the capacity of our model to discern and focus on clinically significant features such as carotid plaques, thereby making it a valuable tool for automated diagnosis and feature extraction. The results suggest that further integration of Grad-CAM into our deep learning model has the potential to enhance the interpretability and transparency of these predictive networks, an attribute of utmost importance in the application of deep learning in the medical imaging domain.

In our study, the AUC of the Modeling for detecting carotid plaques was 0.093 higher than that of the Modeling for assessing plaque stability, with a 11.75% higher sensitivity and a 11.94% higher specificity on the internal test. On the external test, the AUC of the Modeling for detecting carotid plaques was 0.093 higher than that of the Modeling for assessing plaque stability, with a 26.78% higher sensitivity and a 11.94% higher specificity. In classification tasks, the granularity of classification is a key factor that affects classification performance. The analysis suggests that in the two main tasks mentioned in this article, the task of plaque detection only requires a small number of features to achieve good results because the goal is simply to determine whether a plaque exists in the carotid artery. However, for the task of classifying the stability of carotid plaque, a more detailed classification of plaque types is required, and the model needs to learn deeper features for each type. This necessitates more parameters and stronger feature extraction capabilities in the model, which is a challenging task in data-limited scenarios. Consequently, the AUC and other indicators for this model are lower than for coarse-grained tasks. Therefore, we adopted dual-input BCNN network for identifying the stability of carotid plaques. To our knowledge, this model has advancements in recognizing carotid plaque ultrasound images and has not been reported previously.

Our study still has some limitations, the algorithm should be validated and further optimized in larger patient samples, such as through the integration of a transformer-based network. It may be useful to extend the algorithm to the assessment of lipid nuclei in unstable plaques, extent of calcification, or size of ulcerated plaque craters. These features have been linked to the risk of stroke. In future work, we will expand the external validation to include a wider range of locations and populations, which would make the findings more universally applicable. Another useful addition would be a study of how the model performs over time with the same patients, to assess its consistency and reliability.

## 5 Conclusion

In conclusion, we present a combined BCNN-ResNet algorithm that shows superior performance compared to other deep learning methods in detecting carotid plaques and assessing their stability. The BCNN network demonstrates advanced capabilities in recognizing carotid artery ultrasound images. Our BCNN-network outperforms previous models in determining the presence of carotid plaques and identifying their stability. The application of our algorithm could potentially streamline clinical workflows, facilitate clinical screening for carotid artery disease, and contribute to the prevention of stroke.

## Data availability statement

The original contributions presented in the study are included in the article/Supplementary material, further inquiries can be directed to the corresponding authors.

## Ethics statement

The study was approved by the Ethics Review Board at Shanghai Eighth People’s Hospital (approval 2022-015-09-02) and the Xinhua Hospital affiliated with Dalian University (approval 2022-100-01). The studies were conducted in accordance with the local legislation and institutional requirements. Written informed consent for participation was not required from the participants or the participants’ legal guardians/next of kin because this is a retrospective study, we retrospectively analysed the collection of images and information needed for the modelling process. Written informed consent was not obtained from the individual(s) for the publication of any potentially identifiable images or data included in this article because this is a retrospective study, we retrospectively analysed the collection of images and information needed for the modelling process.

## Author contributions

LH: Data curation, Formal analysis, Funding acquisition, Investigation, Methodology, Project administration, Resources, Supervision, Validation, Visualization, Writing – original draft, Writing – review & editing. ZY: Conceptualization, Data curation, Investigation, Methodology, Software, Supervision, Visualization, Writing – original draft. YuW: Conceptualization, Data curation, Formal analysis, Software, Writing – review & editing. WC: Conceptualization, Data curation, Investigation, Methodology, Project administration, Software, Supervision, Visualization, Writing – review & editing. LD: Data curation, Formal analysis, Investigation, Methodology, Software, Validation, Writing – review & editing. YiW: Formal analysis, Investigation, Validation, Writing – review & editing. WY: Data curation, Formal analysis, Investigation, Writing – review & editing. XL: Data curation, Formal analysis, Investigation, Writing – review & editing. YZ: Conceptualization, Data curation, Project administration, Supervision, Validation, Writing – review & editing. YH: Project administration, Supervision, Validation, Writing – review & editing. ES: Funding acquisition, Project administration, Resources, Supervision, Writing – original draft, Writing – review & editing.
