# Supplementary material for: A deep learning algorithm to identify carotid plaques and assess their stability
Source: Front Artif Intell. 2024 Jun 17;7:1321884. doi: 10.3389/frai.2024.1321884 (PMC11215125; doi:10.3389/frai.2024.1321884)
Supplement: Supplementary file 1 [file Image_1.pdf]

supplementary materials

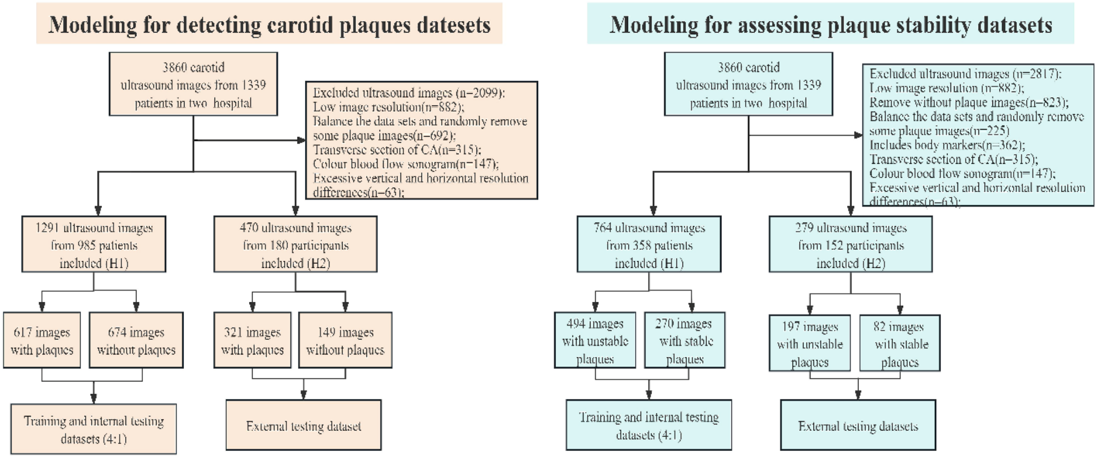

Supplementary Figure 1. Patients and images enrollment flowchart. H1, Shanghai Eighth People's Hospital; H2, Xinhua Hospital affiliated with Dalian University;

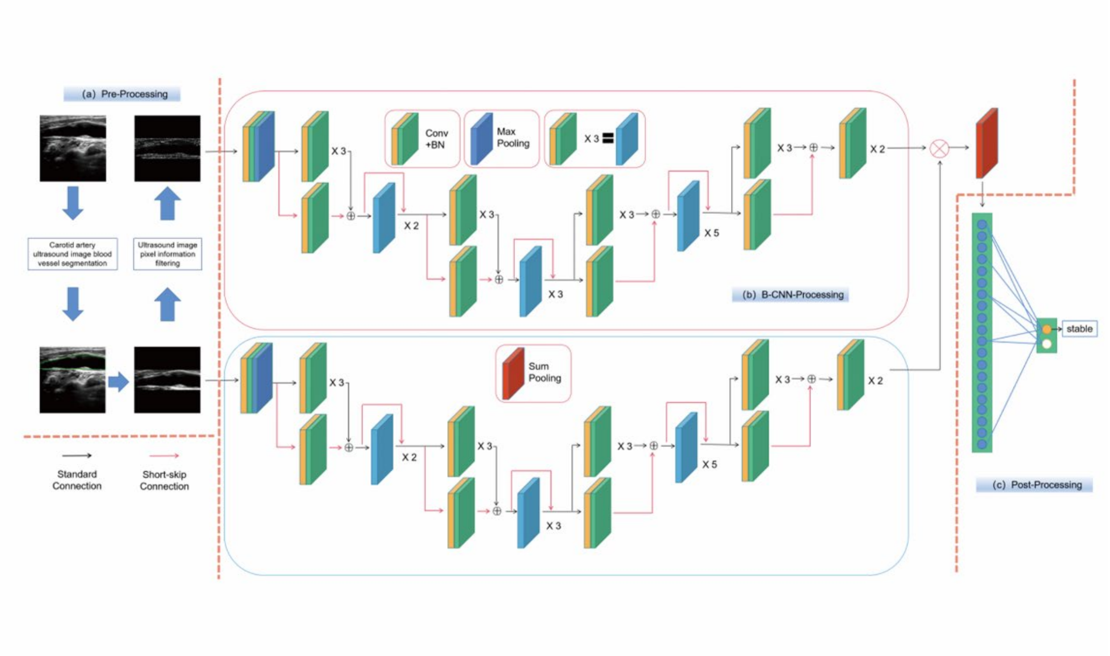

Supplementary Figure 2. A detailed processing flowchart of the proposed Dual-input BCNN Res-Net50

Supplementary Table 1. Comparison of the BCNN Res-Net and other Res-Net architectures for assessing plaque stability in the plaque stability dataset

Algorithm	AUC (95%CI)	TP	FP	Accuracy (%)	Sensitivity (%)	Specificity (%)
ResNet34	0.857 (0.886, 0.814)	89	18	82.30	90.82	67.27
ResNet50	0.860 (0.891, 0.814)	93	20	83.66	94.89	63.63
ResNet101	0.859 (0.891, 0.824)	85	14	82.40	86.70	74.50
Single-input BCNN-ResNet34	0.864 (0.908, 0.840)	82	13	81.05	83.67	76.36
Dual-input BCNN-ResNet34	0.868 (0.908, 0.840)	87	14	83.66	88.78	74.54
Single-input BCNN-ResNet50	0.878 (0.908, 0.840)	86	12	84.31	87.76	78.18
Dual-input BCNN-ResNet50	0.896 (0.922, 0.865)	80	7	83.66	81.63	87.27

AUC, area under the receiver operating characteristic curve; CI, confidence interval; FP, false positive; TP, true positive
